# Reduced spatial resolution MRI suffices to image and quantify drought induced embolism formation in trees

**DOI:** 10.1186/s13007-021-00732-7

**Published:** 2021-04-06

**Authors:** Marco Meixner, Petra Foerst, Carel W. Windt

**Affiliations:** 1 Chair of Process Systems Engineering, Technical University Munich, Munich, Germany; 2 IBG-2: Plant Sciences, Forschungszentrum Jülich, Jülich, Germany

**Keywords:** Magnetic resonance imaging, MRI, NMR, Spatial resolution, Xylem, Embolism, Vulnerability curve, Small-scale, Low field

## Abstract

**Background:**

Magnetic resonance imaging (MRI) is uniquely suited to non-invasively and continuously monitor embolism formation in trees. Depending on the MRI method used, quantitative parameter maps of water content and MRI signal relaxation behavior can be generated. The ability to measure dynamic differences in water content and relaxation behavior can be used to detect xylem embolism formation, even if xylem conduits are too small to be spatially resolved. This is especially advantageous when using affordable small-scale low-field MRI scanners. The amount of signal that can be obtained from an object strongly depends on the strength of the magnetic field of the imager’s magnet. Imaging at lower resolutions thus would allow to reduce the cost, size and weight of the MRI scanner and to shorten image acquisition times.

**Results:**

We investigated how much spatial resolution can be sacrificed without losing the ability to monitor embolism formation in coniferous softwood (spruce, *Picea abies*) and diffuse porous beech (*Fagus sylvatica*). Saplings of both species were bench dehydrated, while they were continuously imaged at stepwise decreasing spatial resolutions. Imaging was done by means of a small-scale MRI device, utilizing image matrix sizes of 128 × 128, 64 × 64 and 32 × 32 pixels at a constant FOV of 19 and 23 mm, respectively. While images at the lowest resolutions (pixel sizes 0.59 × 0.59 mm and 0.72 × 0.72 mm) were no longer sufficient to resolve finer details of the stem anatomy, they did permit an approximate localization of embolism formation and the generation of accurate vulnerability curves.

**Conclusions:**

When using MRI, spatial resolution can be sacrificed without losing the ability to visualize and quantify embolism formation. Imaging at lower spatial resolution to monitor embolism formation has two advantages. Firstly, the acquisition time per image can be reduced dramatically. This enables continuous imaging at high time resolution, which may be beneficial to monitor rapid dynamics of embolism formation. Secondly, if the requirements for spatial resolution are relaxed, much simpler MRI devices can be used. This has the potential to make non-invasive MR imaging of embolism formation much more affordable and more widely available.

**Supplementary Information:**

The online version contains supplementary material available at 10.1186/s13007-021-00732-7.

## Background

During the last couple of decades two non-invasive imaging methods emerged that allow the visualization and quantification of embolism formation and spread in plant xylem: High-resolution computed tomography (HRCT, also referred to as micro-CT) and MRI. For both imaging modalities methods have been developed to determine the percentage loss of xylem conductivity (PLC) due to the cavitation of xylem conduits. This is done by measuring, directly or indirectly, the amount of conducting xylem area that is lost.

HRCT currently is the most widely used method, known for the excellent spatial resolution it affords. It is capable of resolving individual xylem conduits, even in tree species with small conduit diameters. This allows to determine what conduits were filled before dry-down, which conduits cavitate during dry-down, and to estimate their conductivity (and loss thereof) on the basis of their diameters, in combination with the Hagen-Poiseuille law. This has been done for eucalyptus [1], grapevine [2]and walnut [3]. An important drawback of HRCT, however, is the use of ionizing radiation. Depending on the dosage, it may damage living tissue [4], disrupt cellular function [5] and cause growth inhibition [6]. Therefore, while it is an excellent tool to visualize embolized conduits in high spatial detail, it is not an ideal choice to monitor dynamic processes that require repeated or long-term imaging with large numbers of exposures [7].

For the latter purpose MRI, on the other hand, is especially well suited. It does not rely on the absorption of ionizing radiation by the sample, but is based on the principle of nuclear magnetic resonance (NMR). The energy deposition that is associated with MRI of small samples such as plant stems is negligible, especially at low magnetic fields. Therefore, samples can be imaged non-invasively, for periods of months and with a virtually unlimited number of image acquisitions. Examples of such-long term experiments are imaging of flow and water distribution in a tree stem over a period of multiple seasons [8] or imaging of sap flow towards a developing tomato truss [9]. Using the appropriate hardware and imaging pulse sequences, parameter maps can be obtained that quantitatively represent NMR parameters such as amplitude (A) and T_2_ [10]. The amplitude maps acquired in the current study thus linearly and quantitatively reflect water content, and are not merely weighted by it (for further details see [10, 11]).

MRI usually cannot match the resolution offered by HRCT. The resolution that can be achieved by means of MRI is limited by the size of the sample, which usually dictates the smallest field of view (FOV) that can be used, and the number of pixels that can be acquired in that FOV. Reducing the FOV to zoom in on parts of an object in most cases is not possible, as signal from parts of the object that are outside of the FOV would fold back in, corrupting the image. The number of pixels (voxels) that can be encoded in a FOV is then defined by the strength and homogeneity of the magnetic field, as well as the strength of the imaging gradients, but almost never is chosen higher than 512 × 512. For plants matrix size typically is 256 × 256 or lower (for a more detailed explanation of the tradeoffs involved, please see the theory section). This means that in practice, even for samples measuring no more than 10 mm in diameter, it will be hard to exceed an in-plane resolution of 40 × 40 µm. MRI thus can only resolve xylem conduits in species or organs with wide vessels, as has been demonstrated for woody lianas [12], grapevine [2, 14–15], cucumber [16] and roots of maize [17].

To circumvent the problems imposed by this resolution limit, various techniques were developed to detect xylem embolism formation indirectly. In plants, the MR signal almost exclusively originates from protons of water molecules. This fact can be used to generate parameter maps that quantitatively reflect water content, relaxation behavior, diffusion or flow, or parameter maps that are merely weighted by one or more of these parameters. PLC has been calculated or estimated on the basis of parameter maps in a number of ways. One approach is to base PLC on the change in pixel brightness of water content weighted MR amplitude images [18]. PLC values have also been derived from binarizing various types of MR parameter maps. In the binarization approach the loss of xylem conductivity is estimated from the decline in the number of pixels above a chosen threshold, plotted in dependence of the xylem water potential. Binarization has been applied to different types of MR parameter maps, such as signal intensity maps [19–20] or quantitative water content maps [14]. In Meixner et al. [11] we obtained PLC values of beech saplings on the basis of binarizing A*T_2_ product images, obtained by pixel-wise multiplication of water content images (A) and signal relaxation time images (T_2_). In A*T_2_ product images even low numbers of filled xylem vessels per pixel easily became detectable, as well as their disappearance due to cavitation; and the binarization of A*T_2_ product images resulted in accurate xylem vulnerability curves. Using this approach, detection of embolism formation and the quantification of xylem vulnerability curves could be done, despite the fact that pixels were five times larger than the average conduit diameter in beech.

There are two important benefits that may be had by imaging xylem dry-down at a lower than usual spatial resolution: MRI hardware can be scaled down in size and weight, and images can be acquired more rapidly. We recently demonstrated that imaging embolism formation is possible, even when using a mobile small-scale, low-field MRI scanner [11, 21]. The imagers used in these cases were based on a 16 kg permanent magnet. Integrated onto a trolley, the whole system weighed 45 kg and could easily be handled by a single person, even in the field [21]. While this system can already be considered mobile, reducing spatial resolution would enhance mobility even further. If resolution is reduced, a lower magnetic field strength suffices to achieve the same signal-to-noise ratio (SNR), allowing lighter and cheaper permanent magnets to be used. A reduction in resolution would also allow the use of lighter and less powerful gradient amplifiers and therefore permit the development of low-cost imaging equipment that is even more lightweight and mobile. Another advantage when reducing spatial resolution is the ability to image more rapidly, which allows the acquisition of more PLC values per unit time, permitting higher accuracy when fitting vulnerability curves [22]. In addition, high temporal resolution dry-down movies can be generated which allow the investigation of embolism spread within and between annual rings [14, 19], even when this spread proceeds rapidly [18].

In this study we investigate if MRI spatial resolution can be reduced significantly, without losing the ability to detect and quantify embolism formation. To this end, we bench dehydrated spruce (*Picea abies*) and beech (*Fagus sylvatica*) saplings in a small-scale MRI system. During dry-down the plants’ main stems were imaged continuously at three stepwise decreasing spatial resolutions. We tested whether timing and location of embolism formation during dry-down can be monitored and vulnerability curves can be obtained, even if pixel sizes are much (up to thirty times) larger than the average xylem conduit diameter.

### Theory: benefits of imaging at lower spatial resolutions

MR imaging experiments always involve a trade-off between spatial resolution, signal-to-noise ratio (SNR) and imaging time. The benefits of using a lower spatial resolution are illustrated by the following equation [23]:

1 $${\text{SNR}}/{\text{pixel}} \propto {\text{A}}_{{{\text{pix}}}} {\text{B}}_{0} \left( {\frac{{{\text{N}}_{{{\text{avg}}}} }}{{{\text{SW}}/({\text{N}}_{{{\text{fq}}}} *{\text{N}}_{{{\text{ph}}}} )}}} \right)^{{1/2}}.$$

Neglecting signal decay over time due to spin–spin or spin–lattice interactions, Eq. 1 illustrates how increasing the pixel area (A_pix_, i.e. a decrease in matrix size N_fq_ × N_ph_ at constant FOV) can compensate for: (i) the use of magnets of a lower magnetic field strength (B_0_); (ii) imaging with a lower number of averages (N_avg_); or (iii) imaging at a higher spectral width (SW). If lower magnetic fields are permitted, the magnets that provide the main magnetic field can become smaller and lighter. For example, imaging at a matrix size of 32 × 32 pixels instead of at 128 × 128, i.e. lowering spatial resolution by a factor of four while keeping all other imaging parameters the same, would allow the use of a four times weaker magnetic field B_0_, while achieving the same SNR per pixel in the same imaging time.

Alternatively, imaging at lower spatial resolutions would also allow measurements with higher temporal resolution. On the one hand because at lower resolution the number of phase encoding steps (N_ph_) is lower, but also because the number of averages (N_avg_) can be lowered, decreasing image acquisition time. Imaging at higher spectral width (SW) also facilitates mobile imaging in the field. It allows the use of magnets of lower homogeneity and ensures tolerance against the effects of temperature changes and magnet handling on the magnet’s homogeneity during transport and field use [11, 24].

Apart from the static and homogeneous magnetic field (B_0_) magnetizing the sample, MRI also requires switched magnetic field gradients to spatially encode the MR signal. The frequency width of a pixel ($\Delta \nu$) is determined by the gradient strength (G), the pixel width in real space ($\Delta r$) in the encoding direction and the gyromagnetic constant for hydrogen ($\gamma$).

2 $$\Delta \nu = \gamma {\text{G}}\Delta {\text{r}}$$

The minimum value that is acceptable for $\Delta \nu$ is, amongst other factors, determined by the homogeneity of the magnet. The more homogeneous the magnet, the lower $\Delta \nu$ is allowed to be. Small-scale magnets that are exposed to temperature changes and handling, however, require higher $\Delta \nu$ values and therefore more powerful imaging gradients (G). The need for strong and fast gradient pulses in turn limits the mobility of the MR imager. Strong and fast gradient pulses require large and heavy gradient amplifiers. For mobile systems this is particularly relevant, since the gradient amplifiers constitute the largest and heaviest hardware components of an imager, besides the magnet. Keeping all other imaging parameters unchanged, a four times reduction in imaging resolution would lower the gradient strength required by a factor of four. Imaging at lower spatial resolutions (higher values of $\Delta r$) thus also allows for the use of smaller, lighter, cheaper and more mobile gradient amplifiers.

## Materials and methods

### Plant materials and treatment

A potted 3-year-old spruce sapling (*Picea abies*, 82 cm high, 16 mm stem diameter at measurement position) was purchased from a local nursery (Pflanzenwelt Biermann, Trangstedt, Germany), as was a 3-year-old beech sapling (*Fagus sylvatica*, 161 cm high, 16 mm stem diameter at measurement position, Baumschule Leonhard Veith, Merzenich-Golzheim, Germany). Both trees were subsequently grown in a greenhouse at 20 °C day (16 h) and 16 °C night (8 h) temperature, provided with supplemental light (250 W, Philips IP65 metal halogen vapor lamps) and kept well-watered by means of drip irrigation.

**Fig. 1:**
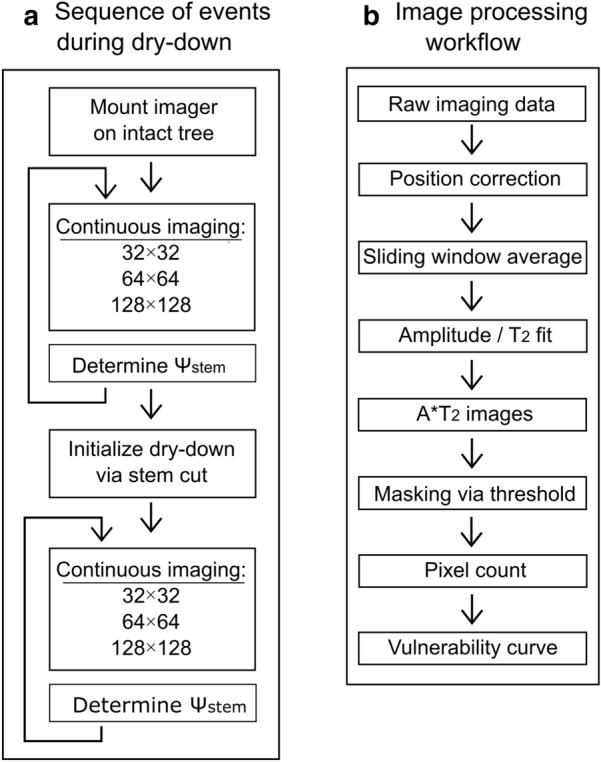
Flow charts of the sequence of events during the dry-down experiment (**a**) and of the image- and data processing workflow (**b**). Images were acquired continuously and at matrix sizes of 32 × 32, 64 × 64 and 128 × 128 pixels

For the dry-down treatment the trees were moved to the lab. MRI scanning was started when the tree was still intact, for the spruce tree 11 h and for the beech tree 9 h prior to the initiation of dry-down, which was done by cutting the stems 5 cm above the soil (for a schematic representation of the experiment, see Fig. 1a). During the entire experiment the trees were illuminated by a sodium vapor lamp (SON-T Agro 400 W, Philips) and kept at a temperature of 22 °C during the day (12 h) and 18 °C at night (12 h). The dry-down experiment was continued until the xylem was completely embolized, which took 47 h in case of spruce and 18 h for beech. The spruce dry-down experiment was performed in the first half of February 2019, the beech dry-down experiment in the middle of June 2019.

### MRI hardware and methods

The MRI scanner comprised a custom, small-scale C-shaped permanent magnet (0.25 T, 16 kg), a custom plane parallel gradient set with a maximum gradient strength of 0.3 T/m and a Kea II spectrometer (Magritek, Wellington, New Zealand), equipped with a built-in 100 W RF amplifier. The gradient set was driven by three BAFPA40 gradient amplifiers (BRUKER, Rheinstetten, Germany). Inside the gradient system a 20 mm solenoidal RF coil with tuning mechanism was mounted. For full details on the system see Meixner et al. [11].

After winding the radio frequency (RF) coil around a Teflon former, placed on the stem (at heights of 37 and 78 cm for spruce and beech respectively), the plants were slid into the probe head of the MRI scanner. Both trees were fixed relative to the imager by means of three metal clamps, positioned below and above the magnet, and near the position where the tree was cut. For both measurements, a glass reference tube with an inner diameter of 1.7 mm, filled with nickel nitrate doped water and a T_2_ value of 112.4 ms, was attached to the stem inside of the RF coil. To minimize external RF noise the magnet was covered with an earthed silver-plated copper mesh.

A multi-spin echo (MSE) pulse sequence was employed as previously described in full detail by Meixner et al. [11], acquiring 64 echoes with an echo time of 4 ms for the first echo and 2.1 ms for all subsequent ones. Excitation was done by means of a slice selective 90°, 500 µs 5-lobe sinc pulse; refocusing by means of hard, 13 µs non-slice selective 180 degree pulses. For all measurements, the spectral width was 100 kHz, the slice thickness 5 mm, the dwell time 10 µs, the number of averages 4 and the repetition time 1.5 s. The field of view (FOV) was 19 × 19 mm for spruce and 23 × 23 mm for beech. Both plants were imaged continuously, acquiring sets of three images of increasing matrix size (32 × 32, 64 × 64 and 128 × 128 pixels) and measurement time (3.5, 7.2 and 14.6 min, see Fig. 1). The resulting in-plane resolutions in mm were 0.59 × 0.59, 0.30 × 0.30 and 0.15 × 0.15 for spruce, and 0.72 × 0.72, 0.36 × 0.36, 0.18 × 0.18 for beech, respectively. By continuously repeating this block of three measurements three time series with different spatial resolutions were acquired, each with the same temporal resolution of 25.3 min (Fig. 1a).

### Data processing

**Fig. 2:**
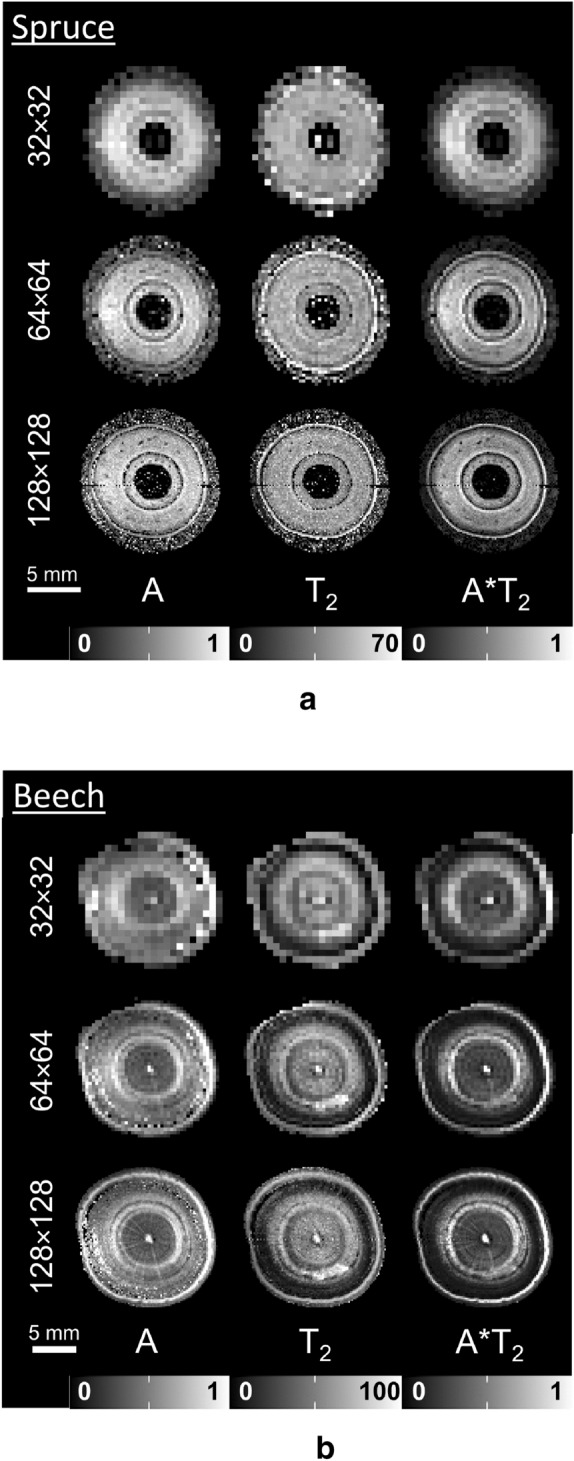
Water content (A), relaxation time (T_2_) and A*T_2_ product images of a well-watered spruce (**a**) and beech (**b**) sapling, acquired at matrix sizes of 32 × 32, 64 × 64, and 128 × 128 pixels. The resulting pixel sizes were 0.59 × 0.59, 0.30 × 0.30 and 0.15 × 0.15 mm for spruce and 0.72 × 0.72, 0.36 × 0.36 and 0.18 × 0.18 mm for beech. The total acquisition times at these respective matrix sizes were 7, 29, and 117 min for spruce and 4, 14 and 59 min for beech. For A and A*T_2_ the values are indicated in arbitrary units (a.u.), T_2_ is indicated in ms

A flow chart of the image processing workflow is given in Fig. 1b. The higher resolution images (matrix sizes 64 × 64 and 128 × 128) were of lower SNR than desired for our purposes (Additional file 1). In order to raise SNR, a sliding window averaging approach was applied, averaging up to eight consecutive measurements of the same spatial resolution. Prior to sliding window averaging all images were corrected for small position changes of the tree during the dry-down. This was done by iteratively shifting each image pixel-wise in the x- and y-direction, looking for the position at which the smallest difference is found between the shifted image and the first image of each time series. This difference was calculated by subtracting the current image in the time series from the first image, then taking the sum total of the absolute values of all pixels. After sliding window averaging all resulting images were phase corrected according to Ma et al. [25]. Subsequently, quantitative water content (A) and signal relaxation (T_2_) maps were obtained by mono-exponentially fitting the echo train of each image of the measurement time series in a pixel-by-pixel manner [26]. Prior to fitting, handmade masks were applied which only included the tree stem. A*T_2_ parameter maps were calculated by multiplying amplitude (A) and T_2_ images, again pixel-by-pixel (Fig. 2).

When generating dry-down movies via sliding window averaging of one, two, four or eight images, fitting errors in the form of erroneous, bright pixels were filtered out. This was done by setting pixels to zero that have double or more, or less than half the value of all four nearest neighboring pixels.

### Calculation of vulnerability curves

To generate vulnerability curves (VCs), the decline in xylem functionality was estimated from A*T_2_ product images and plotted as a function of stem water potential (Ψ_stem_). The latter was measured with a Scholander Chamber (Model 1000, PMS Instrument Company, Albany, USA). Throughout the dry-down, randomly chosen small twigs were carefully bagged with cling foil and aluminum foil (the latter to put the leaves in complete darkness) and left to equilibrate for 15 min. The twigs were then excised, quickly transferred to the instrument and measured. For spruce, 61 water potential values were measured over the course of four days, whereas during the more rapid dry-down of beech 26 water potential values were taken over a period of 26 h.

During the dry-down experiments, the percentage of embolized xylem conducting area was obtained by binarizing A*T_2_ product images. This was done by matching the center of the sliding window temporally to the water potential measurements and counting the number of pixels (P_abv_) above a binarization threshold, the value of which was determined by an optimization approach. Thresholds were iteratively applied to each dry-down series of images and the r^2^ of the sigmodial fits of the resulting dry-down curves compared, until an optimal solution (maximum r^2^) was found. For the three matrix sizes (32 × 32, 238 64 × 64, 128 × 128) this resulted in threshold values of 0.2, 0.1, 0.1 for spruce, and 1.4, 1.3, 1.3 for beech, respectively. After binarization, the P_abv_ values were normalized from 0 to 100% according to $\% _{{{\text{emb}}}} = 100*\frac{{{\text{P}}_{{{\text{initial}}}} - {\text{P}}_{{{\text{abv}}}} }}{{{\text{P}}_{{{\text{initial}}}} - {\text{P}}_{{{\text{min}}}} }}$, with P_abv_ referring to the initial and P_min_ to the minimum number of pixels above the binarization threshold of a time series of images. The VC was obtained by fitting a sigmodial function in dependence of the measured xylem water potential (Ψ_xylem_): $\% _{{{\text{emb}}}} \left( {\Psi _{{{\text{xylem}}}} } \right) = 100/\left( {1 + {\text{exp}}\left( {\frac{{\text{S}}}{{25}}\left( {\Psi _{{{\text{xylem}}}} - \Psi _{{50}} } \right)} \right)} \right)$ [22], with the fit parameters indicating how quickly the dry down progressed through the xylem (slope: S) and the water potential at which 50% of the initially filled xylem was embolized (Ψ_50_). Normalization and sigmodial fitting were performed with Matlab (Mathworks, Natick, USA). Fitting errors were calculated as described by Press [27].

### Microscopy

After the dry-down experiment, the imaged stem pieces were marked, excised and stored in 75% ethanol (after previously equilibrating them in 25% and 50% ethanol for 24 h each). Transverse sections of 20 µm were prepared with a sliding microtome (GSL1, Schenkung Dapples, Zürich, Switzerland) and stained with a 1:1 (v/v) mixture of safranin and astrablue, following Gärtner and Schweingruber [28]. Images of the cross section were obtained with a digital camera (DFC 320, Leica, Cambridge, UK), connected to a light microscope (DM2500, Leica Microsystems GmbH, Wetzlar, Germany).

## Results and discussion

### What xylem anatomical detail can be resolved at decreasing spatial resolution?

In spruce, water filled xylem could easily be distinguished from cambium and bark, even at a pixel size of 0.59 × 0.59 mm (matrix size 32 × 32 in Fig. 2a). Individual year rings, however, were only resolved at a pixel size of 0.30 × 0.30 mm (matrix size 64 × 64) and better. The cambium is the brightest tissue in the T_2_ images, where it showed maximum contrast against the surrounding tissue. The border between cambium and xylem, however, appearing as a black ring both in water content (amplitude, A) and T_2_, was only resolved at pixel sizes of 0.30 × 0.30 mm (matrix size 64 × 64) and 0.15 × 0.15 mm (matrix size 128 × 128). The homogeneous distribution of water inside the filled xylem was similar to that previously imaged in pine stems [20, 29].

In beech, at all pixel sizes the filled xylem could be distinguished from the cambium due to a dark region between cambium and filled xylem (Fig. 2b). Similar to spruce, individual year rings could only be recognized at pixel sizes of 0.36 × 0.36 mm (matrix size 64 × 64) and better. In the microscopic images of beech (Fig. 4b), the sapling showed two complete growth rings, and a narrow region of early wood of a third growth ring inside of the cambium, which had formed during the current year. At all matrix sizes xylem containing filled vessels could be distinguished from non-filled xylem on the basis of higher water contents (40 to 60%), but could be identified even more clearly by long T_2_ (60 to 80 ms) and A*T_2_ values. Filled xylem was not found in all year rings, but only became visible in the middle of the second growth ring. Comparable water content and T_2_ values for filled and embolized xylem in beech were found in Meixner et al. [11]. Merela et al. [30] observed similar water content distribution patterns in the stem of a beech sapling (please note that in this example the amplitude images were water content weighted, but did not reflect water content quantitatively). The absence of filled vessels in the newly formed xylem may indicate that the plant experienced drought stress [31], or had been repotted in spring 2019.

Most of the finer details of the xylem anatomy of both beech and spruce thus were already resolved at a pixel size of 0.30 × 0.30 mm in spruce and 0.36 × 0.36 mm in beech (in both cases, at a matrix size of 64 × 64). Pixel sizes of 0.59 × 0.59 mm in spruce and 0.72 × 0.72 mm in beech (matrix size of 32 × 32) were sufficient to identify filled xylem.

### Spatio-temporal characterization of the xylem dry-down at decreasing spatial resolution

**Fig. 3:**
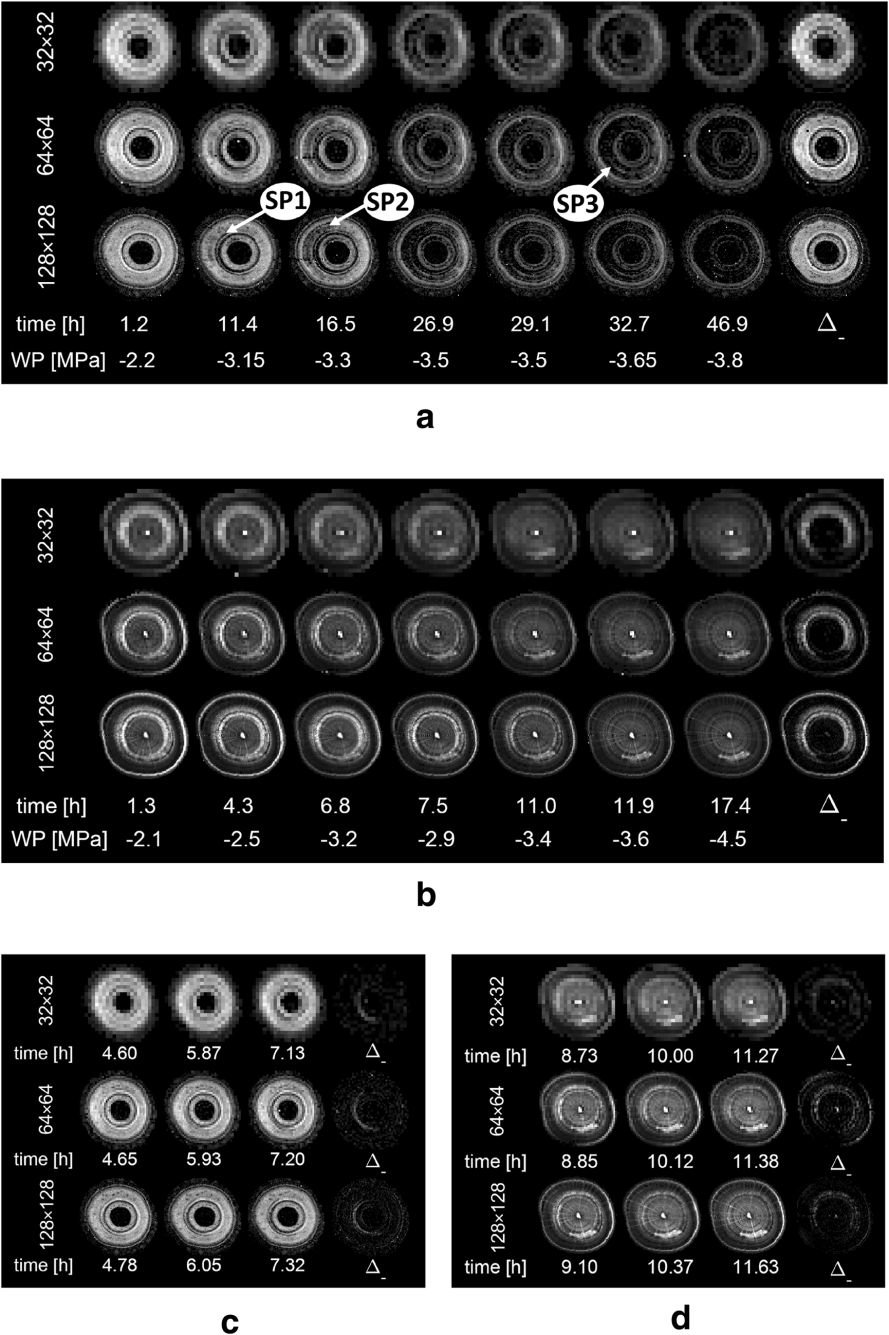
A*T_2_ product maps of xylem embolism formation in spruce (**a**) and beech (**b**). Images were acquired with matrix sizes of 32 × 32, 64 × 64 and 128 × 128 pixels, resulting in pixel sizes of 0.59 × 0.59, 0.30 × 0.30 mm and 0.15 × 0.15 for spruce and 0.72 × 0.72, 0.36 × 0.36 and 0.18 × 0.18 mm for beech). Below the parameter maps desiccation time and water potential (WP) are shown. The concomitant water content and T_2_ images are provided in Additional file 2. Imaging continuously allowed for resolving finer spatio-temporal details of embolism formation. In (**c**) the first emboli forming in spruce are shown, in panel (**d**) the completion of xylem dry-down in beech. In all panels (**a**–**d**), the difference in image intensity between the first and the last image is shown in the rightmost position (${\Delta }_{-}$)

The A*T_2_ product images provided an effective means to monitor dry-down for all three matrix sizes in both xylem anatomies (Fig. 3a, b). In Meixner et al. [11] it was shown that both water content and T_2_ images can be used to detect xylem cavitation in beech, but that T_2_ maps are especially sensitive to it. By multiplying the parameter maps of water content (A) and T_2_, A*T_2_ product maps were obtained, in which regions with filled vessels in beech could be identified even more clearly. In the current study we show that in coniferous wood the same approach is effective as well. The presence of filled xylem conduits gave rise to bright A*T_2_ regions, just like in beech, while dry-down manifested itself as a strong decrease in A*T_2_ values (Fig. 3a). In the following the dry-down of both species was therefore monitored on the basis of A*T_2_ maps.

In the A*T_2_ product maps of spruce, independent of matrix size, the first emboli could be distinguished at a water potential of – 3.15 MPa, 11.4 h after dry-down initiation (Fig. 3a). Embolism formation started in the current annual ring and at the border of the neighboring second ring (Fig. 3a, arrow SP1). From this region emboli spread, first radially and then tangentially within the outer annual ring (arrow SP2), and after 16.5 h emboli also started forming in the neighboring ring. Another detail that was visible at all three spatial resolutions was a narrow strip of xylem that remained hydrated in the outer annual ring, while the xylem tissue surrounding it appeared empty (arrow SP3). After 46.9 h (− 3.8 MPa) nearly all xylem was embolized (Fig. 3a).

In beech, emboli started forming only a few hours after cutting the plant at its base (Fig. 3b). Embolism formation manifested itself in the form of a gradual decline in A*T_2_ pixel intensities. Filled xylem, which was found only in the middle of the second growth ring, cavitated within a short time window, between hour 7.5 and 12.0. Dry-down appeared gradual and spread uniformly over the entire filled xylem, indicating that vessels cavitated in an independent, rather than in a clustered manner. Embolism spread in a tangential and radial pattern, as observed by Meixner et al. [11] within beech, was not detected.

**Fig. 4:**
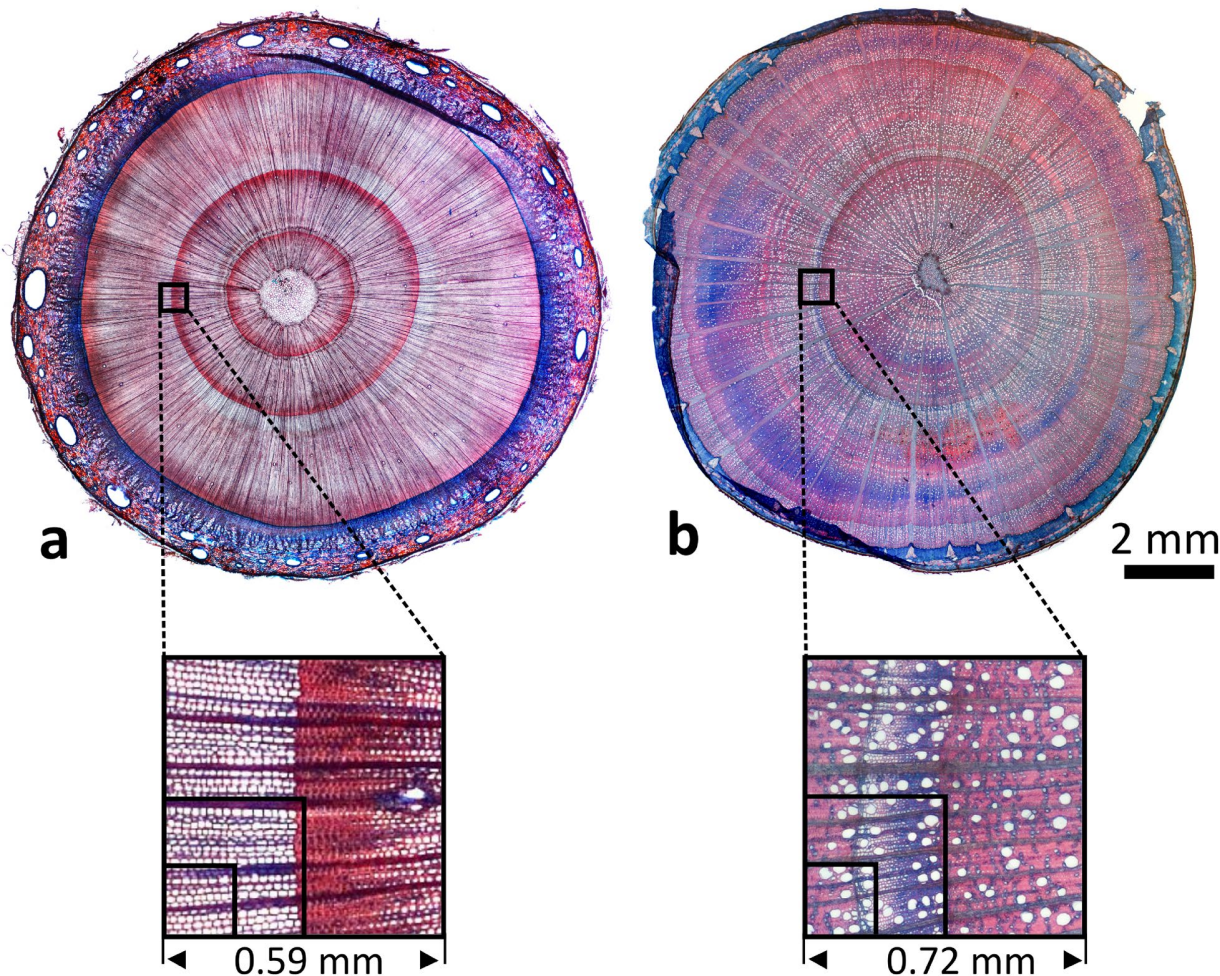
Light microscopy of spruce (*Picea abies*, **a**) and beech (*Fagus sylvatica*, **b**). The thin sections were obtained from the same position where MR imaging was done. Insets illustrate the difference in pixel sizes between the three spatial resolutions (matrix sizes) used in the MR imaging experiments

On the basis of difference images, even at the lowest spatial resolution, it was possible to detect embolism events that play out in a time window of only a few hours (see ${\Delta }_{-}$-maps in Fig. 3c, d). In spruce it was shown that the onset of embolism formation, which took place in the early wood of the outer annual ring, happened between hour 5 and 8 after initiation of the dry-down (Fig. 3c). In beech at the lowest spatial resolution it could be shown that the final phase of the xylem dry-down happened between hour 9 and 12 (Fig. 3d). A striking difference between the sequences of A*T_2_ product images of both species was that in the xylem of spruce almost no intensity was left in the images after complete dry-down, whereas in beech always a significant amount of background signal was left visible. This reflects the differences in xylem anatomy of spruce and beech. In spruce, the xylem for the largest part consists of tracheids, and contains, apart from resin ducts, almost no other tissue types or living cells [32] (Fig. 4a). After cavitation of the tracheid, the amount of remaining water in the tracheid walls was either too little to be recognized, or its T_2_ value was too short to be detected. In beech, to the contrary, the bulk of the xylem consists of narrow fibers with thick walls, interspersed between the vessels (Fig. 4b). This pool of water remained visible and, with an average T_2_ value of ~ 25 ms, was detected in the stem, even when surrounding vessels cavitated.

For an animated illustration of the observations mentioned in this section a tableau of nine videos are provided for both plants as supplementary material (Additional file 3a, 4b).

### Are low resolution images sufficient to obtain xylem vulnerability curves?

**Fig. 5:**
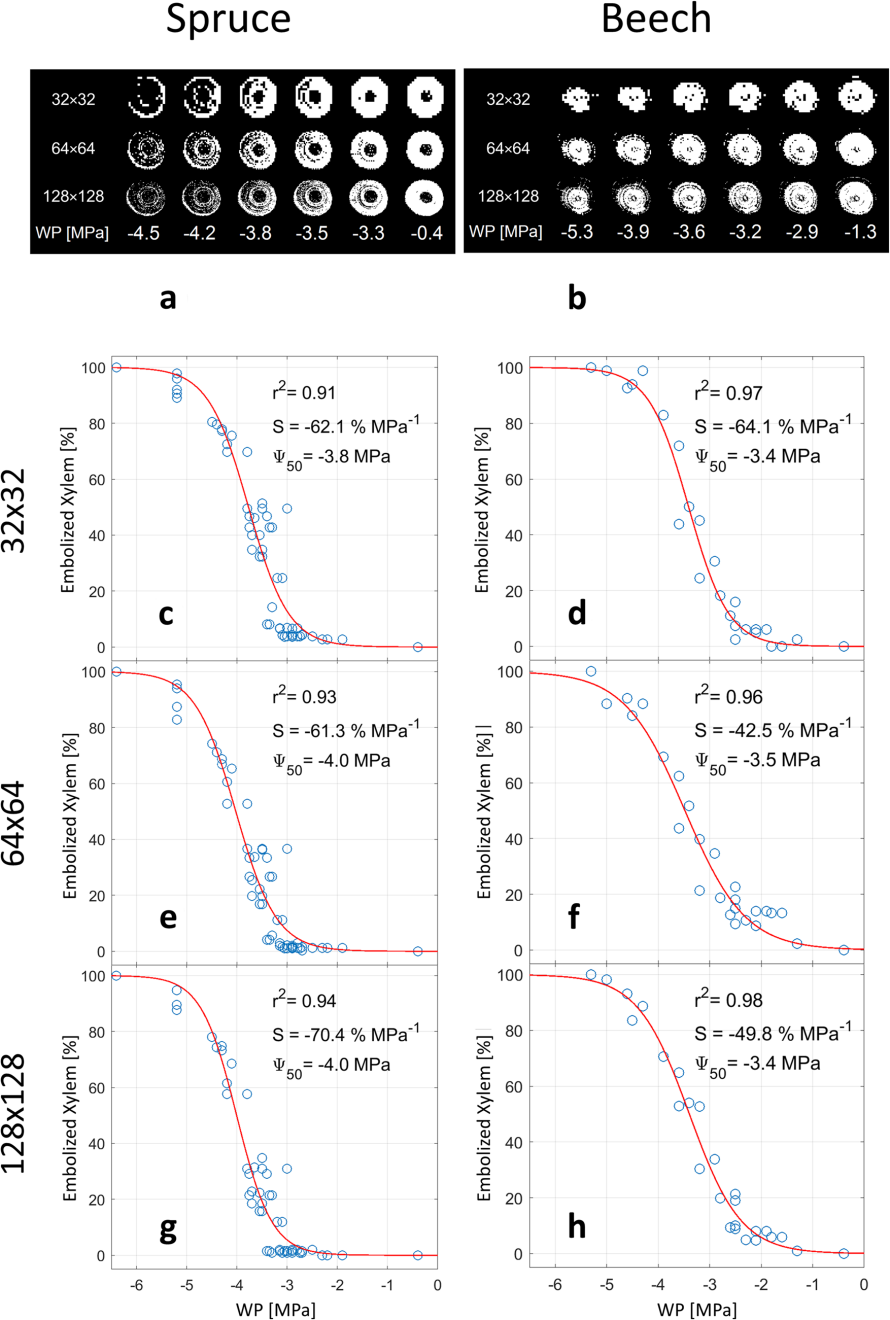
Vulnerability curves (VCs) obtained on the basis of binarization of A*T_2_ product maps. The matrix size of the images was varied (32 × 32, 64 × 64, and 128 × 128, resulting in pixel sizes of 0.59 × 0.59, 0.30 × 0.30 and 0.15 × 0.15 mm for spruce and 0.72 × 0.72, 0.36 × 0.36 and 0.18 × 0.18 mm for beech), while field of view was held constant. A subset of the binarization masks that the VC’s are based on is shown, together with the corresponding water potentials (WP), in (**a**) for spruce and (**b**) beech. The resulting VCs are shown in panels (**c**, **e**, **g**) for spruce; and in panels (**d**, **f**, **h**) for beech

For spruce and beech, xylem vulnerability curves (VCs) were successfully obtained via binarizing A*T_2_ product images of all three spatial resolutions. For both species, the binarization masks at different matrix sizes were consistent in the location and timing where xylem desiccation was observed (Fig. 5a, b). VCs based on those binarization masks showed r^2^ values > 0.90 and yielded comparable Ψ_50_ values. At matrix sizes of 32 × 32, 64 × 64, 128 × 128 for spruce values of − 3.8, − 4.0 and − 4.0 MPa were found, for beech − 3.4, − 3.5 and − 3.5 MPa, respectively (Fig. 5c–h).

The Ψ_50_ values found for beech at the different matrix sizes were in close agreement with one another, as well as with values obtained in other studies on beech saplings (Caquet et al. [33]: − 3.0 to − 4.0 MPa; Aranda et al. [34]: − 3.0 to − 3.7 MPa; Meixner et al. [11]: − 3.2 to − 3.8 MPa) and mature beech trees (Lemoine et al. [35]: − 2.2 to − 3.1 MPa; Herbette et al. [36]: − 3.0 MPa; Schuldt et al. [37]: − 3.3 to − 3.7 MPa; Stojnic et al. [38]: − 2.9 to 3.5 MPa; Tomasella et al. [39]: − 2.5 MPa). For spruce, the Ψ_50_ values obtained in the current study were slightly higher than those obtained in other studies on spruce saplings (Mayr et al. [40]: − 4.38 MPa; Chmura et al. [41]: − 4.27 MPa), but lower or in agreement with values found for mature trees (Cochard [42]: − 3.5 MPa; Tomasella et al. [39]: − 4.01 MPa). Hence, we conclude that xylem dry-down can be quantified accurately on the basis of MR images, even if their spatial resolution is low and pixels are much larger than xylem conduit diameters.

## Conclusions

In this study we demonstrated that MR imaging at low spatial resolution (pixel sizes > 0.5 × 0.5 mm) suffices to detect and image the formation of xylem emboli in spruce and beech, and that these images can be used to quantify embolism formation by means of xylem vulnerability curves. While images with a matrix size of 32 × 32 (pixel sizes of 0.59 to 0.72 mm) were not sufficient to spatially resolve the annual rings or finer details of the stem anatomy, they did permit an approximate localization of embolism formation (e.g. to differentiate older and younger xylem tissue on the basis of their position in the stem).

The ability to obtain meaningful data and vulnerability curves from quantitative but low-resolution images can be utilized in two ways. Firstly, by imaging at lower spatial resolution the acquisition time per image can be reduced dramatically. This enables continuous imaging at high time resolution, which may be beneficial to monitor rapid dynamics of embolism formation, for example during bench dehydration. Secondly, if the requirements for spatial resolution are relaxed, much simpler MRI devices can be used. Such imagers can be based on much weaker and therefore smaller and lighter permanent magnets, as well as on smaller and lighter gradient amplifiers. For example, keeping all other imaging parameters constant, accepting a four times lower imaging resolution allows the strength of the main magnetic field as well as that of the imaging gradients to be reduced by a factor of four, without sacrificing SNR or increasing imaging time. Imaging at reduced spatial resolutions thus has the potential to make MR imaging of embolism formation much more mobile and affordable, further opening up the methodology to the plant hydraulics community.

## Supplementary Information

**Additional file 1.** First echo images of well-watered spruce (a) and . beech (b), acquired with an MSE imaging sequence, illustrating the dependency of image quality and signal-to-noise ratio on matrix size and the number of acquisitions averaged (numAcq). The respective SNR is printed under each image.

**Additional file 2**. Water content (A) and T_2_ maps of progressive xylem embolism formation in spruce (a ,b) and beech (c,d), acquired with matrix sizes of 32 × 32, 64 × 64 and 128 × 128 pixels, and shown in dependence of time and water potential (WP). In all panels (a-d), the difference in image intensity between the first and the last image is shown in the rightmost position (${\Delta }_{-}$).

**Additional file 3.** Animated illustration of the xylem dry-down of spruce (a). Water content (A), T_2_ and A*T_2_ maps were acquired at image matrix sizes of 32 × 32, 64 × 64 and 128 × 128 pixels. In the upper right corner dry down time and water potential are shown.

**Additional file 4.** Animated illustration of the xylem dry-down of beech (b). Water content (A), T_2_ and A*T_2_ maps were acquired at image matrix sizes of 32 × 32, 64 × 64 and 128 × 128 pixels. In the upper right corner dry down time and water potential are shown.

## Data Availability

The datasets used and/or analysed during the current study are available from the corresponding author on reasonable request.
