# Supplementary figures and images for: Reduced spatial resolution MRI suffices to image and quantify drought induced embolism formation in trees
Source: Plant Methods. 2021 Apr 6;17:38. doi: 10.1186/s13007-021-00732-7 (PMC8025330; doi:10.1186/s13007-021-00732-7)

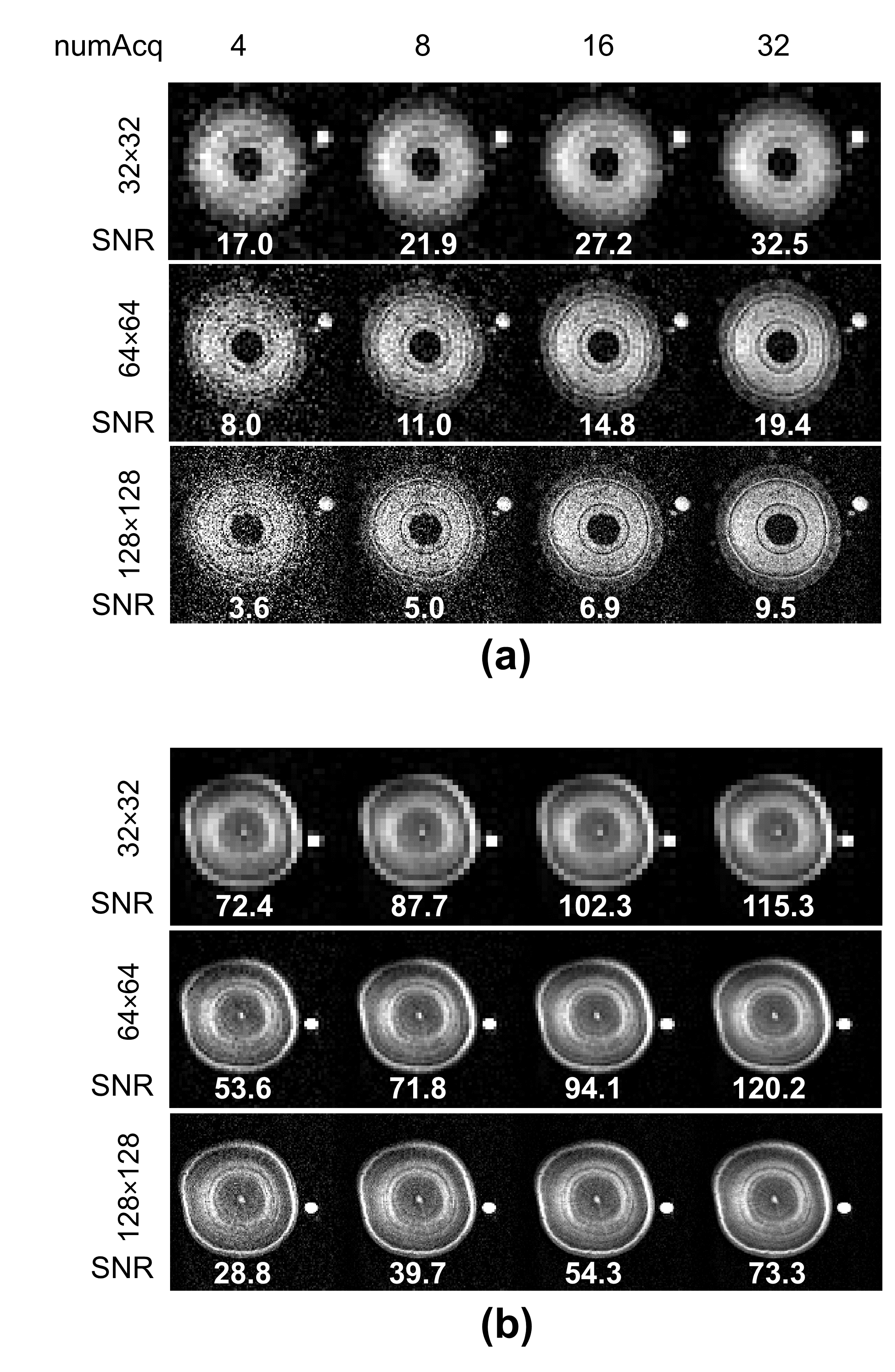

Supplement: Supplementary file 1 — Additional file 1. First echo images of well-watered spruce (a) and . beech (b), acquired with an MSE imaging sequence, illustrating the dependency of image quality and signal-to-noise ratio on matrix size and the number of acquisitions averaged (numAcq). The respective SNR is printed under each image. [file 13007_2021_732_MOESM1_ESM.tif]

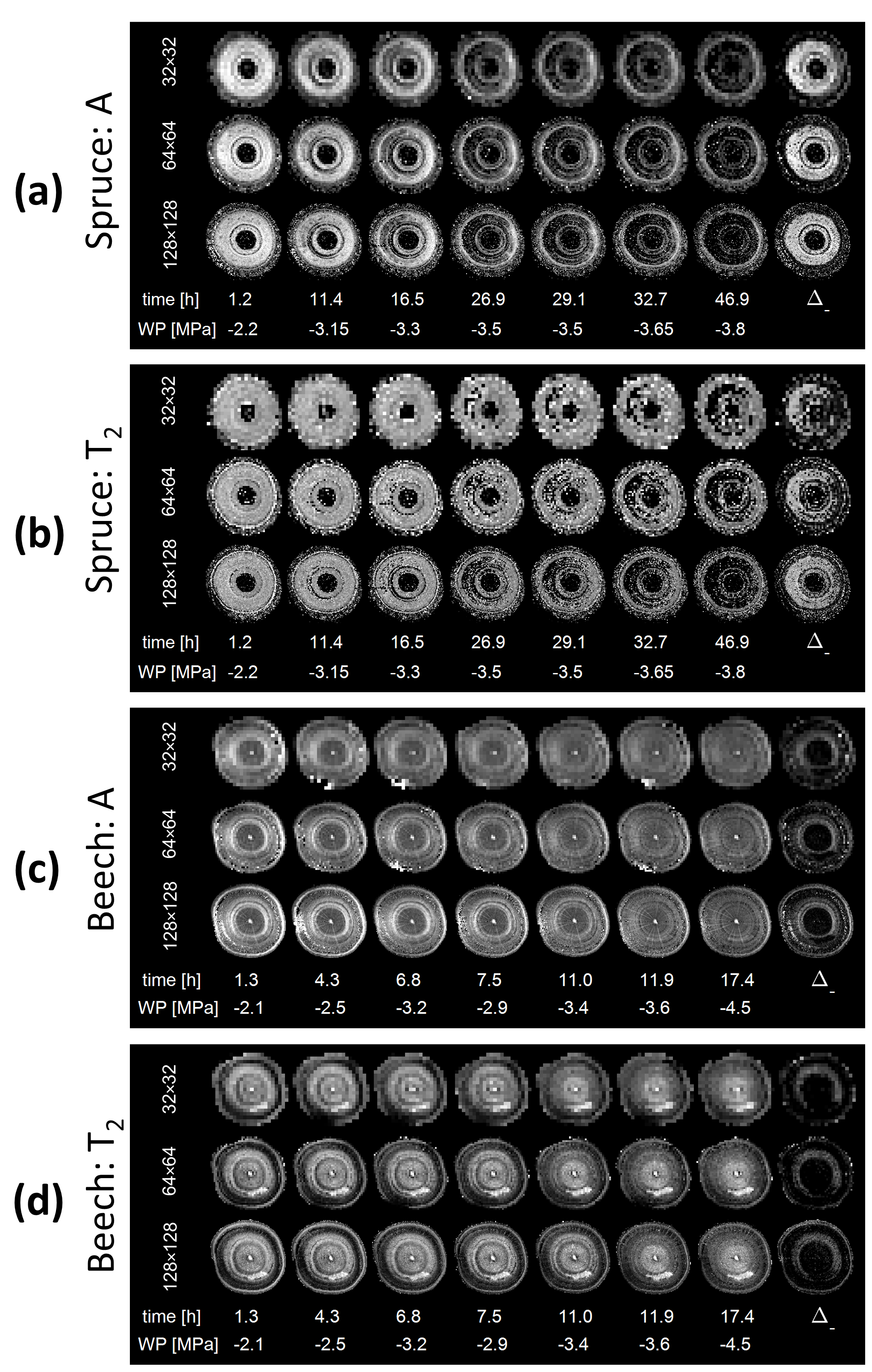

Supplement: Supplementary file 2 — Additional file 2. Water content (A) and T2 maps of progressive xylem embolism formation in spruce (a ,b) and beech (c,d), acquired with matrix sizes of 32 × 32, 64 × 64 and 128 × 128 pixels, and shown in dependence of time and water potential (WP). In all panels (a-d), the difference in image intensity between the first and the last image is shown in the rightmost position (${\Delta }_{-}$Δ-). [file 13007_2021_732_MOESM2_ESM.tif]
